# Microbiota–Gastric Cancer Interactions and the Potential Influence of Nutritional Therapies

**DOI:** 10.3390/ijms25031679

**Published:** 2024-01-30

**Authors:** Pauline Raoul, Valeria Maccauro, Marco Cintoni, Emidio Scarpellini, Gianluca Ianiro, Antonio Gasbarrini, Maria Cristina Mele, Emanuele Rinninella

**Affiliations:** 1 Clinical Nutrition Unit, Department of Medical and Abdominal Surgery and Endocrine-Metabolic Sciences, Fondazione Policlinico Universitario Agostino Gemelli IRCCS, 00168 Rome, Italy; paulineceline.raoul@policlinicogemelli.it (P.R.); marco.cintoni@unicatt.it (M.C.); mariacristina.mele@unicatt.it (M.C.M.); 2 School of Specialization in Internal Medicine, Catholic University of the Sacred Heart, 00168 Rome, Italy; valeriamaccauro@gmail.com; 3 Research and Training Center in Human Nutrition, Catholic University of the Sacred Heart, 00168 Rome, Italy; antonio.gasbarrini@unicatt.it; 4 Translationeel Onderzoek van Gastro-Enterologische Aandoeningen (T.A.R.G.I.D.), Gasthuisberg University 11 Hospital, KU Leuven, Herestraat 49, 3000 Leuven, Belgium; emidio.scarpellini@med.kuleuven.be; 5 Digestive Disease Center (CEMAD), Department of Medical and Abdominal Surgery and Endocrine-Metabolic Sciences, Fondazione Policlinico Universitario Agostino Gemelli IRCCS, 00168 Rome, Italy; gianluca.ianiro@unicatt.it; 6 Department of Translational Medicine and Surgery, Catholic University of the Sacred Heart, 00168 Rome, Italy

**Keywords:** gastric cancer, oral microbiota, gut microbiota, gastric mucosa, diet, nutritional therapies

## Abstract

Gastric cancer (GC) is one of the most common causes of cancer deaths, and GC treatments represent a large area of research. Although initially regarded as a sterile organ and unsuitable for microbial communities, the discovery of *Helicobacter pylori* made us realize that some microbes can colonize the stomach. In recent years, growing interest in gastric bacteria has expanded to the gut microbiota and, more recently, to the oral microbiota. Indeed, the oral–gastric–gut microbiota axis may play a crucial role in maintaining homeostasis, while changes in microbiota composition in GC patients can influence clinical outcomes. On the one hand, the microbiota and its metabolites may significantly influence the progression of GC, while anti-GC treatments such as gastrectomy and chemotherapy may significantly impact the oral–gastric–gut microbiota axis of GC patients. In this context, the role of nutritional therapies, including diet, prebiotics, and probiotics, in treating GC should not be underestimated. Wit this review, we aim to highlight the main role of the gastric, oral, and gut microbiota in GC onset and progression, representing potential future biomarkers for early GC detection and a target for efficient nutritional therapies during the course of GC.

## 1. Introduction

Gastric cancer (GC) is the third leading cause of cancer-related mortality worldwide [1]; corresponding to the fifth highest incidence in the world, it is estimated to cause over one million new cases worldwide each year [2]. Five-year GC survival rates are estimated at almost 30% in most western countries due to late-stage diagnosis [3]. GC is initiated insidiously, and the early symptoms are atypical. Thus, most patients have already progressed to advanced stages upon diagnosis, making their treatment challenging. GC is a multifactorial disease affected by various genetic and environmental factors, including *Helicobacter pylori* (*H. pylori*) infection, lifestyle, socioeconomic factors, dietary behavior, and aging [4].

Until recently, the gastric environment was considered sterile, probably due to increased acidity, but emerging data have revealed that there is a broad range of microorganisms in the stomach with a density of 10 to 1000 colony-forming units/g. The gastric microbiota comprises bacteria ingested mainly through the respiratory tract and secondarily from the intestine by transpyloric biliary reflux. The predominant phyla in the gastric mucosa consist of acidity-resisting bacteria such as Actinomycetota, Bacillota, Bacteroidota, and Pseudomonadota, among more than one hundred types, the most important of which is *H. pylori* [5]. Since 1994 it has been universally accepted that *H. pylori* is the main driver of the precancerous cascade and the most important etiological factor for GC, estimated between to be involved in between 74.7% and 90% of new non-cardia GC cases [6].

Furthermore, recent studies have focused not only on the gastric microbiota but also on the oral microbiota. Indeed, the oral microbiota exhibits the second highest level of alpha diversity following that of the gut, including over 700 species of bacteria, over 100 species of fungi, and protozoa. The latter include *Entamoeba gingivalis* and *Trichomonas tenax.* Phyla such as Actinomycetota, Bacillota, Pseudomonadota, Bacteroidota, and Saccharibacteria are well represented in healthy oral microbiota. An imbalanced microbiota, known as dysbiosis, is associated with intraoral diseases such as dental caries, gingivitis, and periodontitis, as well as with different neoplasms such as gastrointestinal cancers [7,8].

Finally, in recent years, the association between the gut microbiota and GC has gradually attracted researchers’ attention. The gut microbiota may be involved in carcinogenesis, mainly by modulating immune responses, and, consequently, in the mechanisms of cancer treatment, potentially affecting cancer treatment responses [9].

Concomitantly to GC treatment like surgery or chemotherapy, interest is increasing in nutritional therapy to improve patients’ nutritional status, adherence to therapies, and quality of life. European nutritional guidelines recommended nutritional support for GC patients undergoing surgery, as well as those with unresectable disease; surgery may be performed in combination with oral, enteral, and parenteral nutrition [10].

The objective of this review is to highlight the main role of the gastric, oral, and gut microbiota in GC onset and progression, representing potential future biomarkers for early GC detection and a target for efficient nutritional therapies during the course of GC.

## 2. Gastric Microbiota and Gastric Carcinogenesis

### 2.1. Mutual Relationships between Chronic Gastric Inflammatory Response to H. pylori and Gastric Microbiota Dysbiosis

A normal gastric microbiota is composed of Pseudomonadota (*H. pylori* belongs to this phylum), Bacillota, Actinomycetota, Bacteroides, and Fusobacteria, which are the most abundant phyla. *H. pylori* has inhibitory effects on the colonization of other bacteria, harboring a significantly lower diversity of such bacteria in the stomach. On the contrary, the gastric microbiota in *H. pylori*-negative patients was found to be predominated by other bacterial strains, including Pseudomonadota (52.6%), Bacillota (26.4%), Bacteroidota (12%), and Actinomycetota (6.4%); the most common genera included *Gemella*, *Prevotella*, and *Streptococcus* [11].

*H. pylori* has direct oncogenic potential in GC due to its oncoprotein cytotoxin-associated gene A (CagA), which destabilizes cellular junctions by disrupting the E-cadherin–β-catenin complex and activates proliferating pathways as an ERK-MAP kinase cascade, leading to cellular morphological transition to the “hummingbird” phenotype [12]. In addition, *H. pylori* can facilitate gastric cells’ oncogenic transformation via its vacuolating cytotoxin A (VacA), which interferes with normal autophagy, thereby promoting abnormal cell survival [13].

Given that *H. pylori* also creates a premalignant environment of atrophy and intestinal metaplasia, the subsequent alteration in the gastric microbiota seems to play a crucial role in gastric tumorigenesis itself. Consequently, successful *H. pylori* eradication seems to be essential for the restoration of a balanced gastric microbiota and the prevention of gastric carcinogenesis—at least in the primary stages [14]. It has also been noted that the activity of gastritis demonstrates a close relationship with either *H. pylori* or with other pathogenic phyla, as it increases the abundance of Bacteroidota, Bacillota, or Pseudomonadota, favoring GC-related dysbiosis [15]. *H. pylori* may modify the gastric environment, paving the way for the growth of a dysbiotic gastric bacterial community. In this regard, *H. pylori* eradication may reverse gastric dysbiosis to a similar level in uninfected patients, exerting beneficial effects on the gut microbiota and achieving increased probiotic and putative downregulation of drug resistance. More specifically, successful *H. pylori* eradication was reported to significantly inhibit dysbiosis, and treatment failure was associated with an increased dysbiosis rate comparable to that of active *H. pylori* infection [16]. However, the exact background of the interaction between sustained inflammation and genotoxicity damage has still not been evaluated [15]. In a recent study, a possible explanation of this mutual relationship was evidenced: chronic gastric inflammatory response to *H. pylori* may modify the gastric environment, mainly by reducing gastric acidity, leading to the overgrowth of a dysbiotic gastric bacterial community. In this way, successful *H. pylori* eradication has been shown to significantly reverse gastric dysbiosis to a similar level as in uninfected patients, although the dysbiosis rate of such patients remained higher than the non-*H. pylori* control. Nonetheless, treatment failure was associated with an increased dysbiosis rate comparable to that of active *H. pylori*. More intense dysbiosis was also found to be associated with progress from gastritis to atrophy and GC [16].

In antral-predominant gastritis, the production of gastric acid is increased, which is associated with a high risk of the development of duodenal ulcer disease, whereas it is protective against GC development. In contrast, corpus-predominant gastritis leads to reduced production of gastric acid and may lead to atrophic gastritis, a condition associated with an increased risk of developing GC. *H. pylori* exerts a direct inflammatory effect on the mucosal surface of the stomach, variably affecting the production of mucin, which, in turn, affects the composition of the nearby microbiota, resulting in gastric dysbiosis. A reduced capacity for gastric acid secretion allows for the survival and proliferation of other microbes that are normally killed by acidic environments. This may be the backbone of GC development, given that during the last stage of gastric malignancy, oral or intestinal-type bacteria are predominantly discovered, which is not observed in premalignant conditions (e.g., chronic gastritis and atrophy) where *H. pylori* abundance is evident. Whether this phenomenon is due to tumor-related mucin-type differentiation, possibly resulting in GC-related microbiota, must be elucidated [17].

### 2.2. Close Association between Gastric Microbiota Dysbiosis and GC

A study examined the composition of the microbiota of the gastric mucosa in intestinal-type GC, suggesting that bacterial diversity decreased at the genus level as patients progressed from superficial gastritis to intestinal metaplasia and GC. The authors also described an increased abundance of *Lactobacillus coleohominis* and *Lachnospiraceae* in GC patients, indicating the presence of microbial dysbiosis in gastric carcinogenesis [18]. Another cohort study confirmed the hypothesis of procarcinogenic gastric dysbiosis, underlining that *Parvimonas micra*, *Dialister pneumosintes*, *Slackia exigua*, *Peptostreptococcus stomatis*, *Prevotella intermedia*, *Fusobacterium nucleatum*, *Prevotella oris*, and *Catonella morbi* were significantly enriched in the GC microbiota compared to precancerous stages [19]. In parallel, a Portuguese study reported that the GC microbiota demonstrated reduced microbial diversity; reduced *Helicobacter* abundance; and over-representation of bacterial genera belonging to intestinal commensals of Pseudomonadota, Bacillota, and Actinomycetota phyla. Among Pseudomonadota, an overgrowth of the genera *Phyllobacterium* and *Achromobacter* and the families *Xanthomonadaceae* and *Enterobacteriaceae* was reported. Additionally, Lactobacillaceae family member, *Clostridium* and *Rhodococcus* were also significantly more abundant in gastric carcinoma, whereas *Helicobacter*, *Neisseria*, *Prevotella*, and *Streptococcus* were most abundant in the microbiota of patients with chronic gastritis. In addition to oral cavity bacterial strains, intestinal mucosal commensal, such as *Citrobacter*, *Clostridium*, Lactobacillaceae family, *Achromobacter*, and *Rhodococcus*, were reported to be significantly increased in GC samples compared with chronic gastritis samples. This observation is compatible with the hypothesis that during carcinogenesis, changes in the stomach’s mucosa that lead to decreased acid secretion allow for the growth of bacteria with nitrosative functions, exerting a major genotoxic potential. In these bacteria, the nitrate reductase enzyme, whose function is degradation of nitrate into nitrite and nitrite into nitric oxide, appears to be overexpressed, resulting in gastric cell DNA damage [20]. A case–control study conducted by Gunathilake showed that patients with GC had higher relative abundances of *Helicobacteraceae*, *Propionibacteriaceae*, and *Prevotellaceae* than healthy subjects at the family level, with overall relative abundances of *Helicobacter*, *Propionibacterium*, and *Prevotella* at the genus level, whereas the relative abundance of *Lactococcus lactis* (*L. lactis*) was higher in the healthy controls than in infected patients. Moreover, the Shannon index was significantly higher in the controls than in the patients, as *H. pylori*-positive carriers were found to have higher abundances of *Spirochetes*, *Acidobacteria*, and non-*Helicobacter Pseudomonadota* and comparatively lower abundances of *Actinomycetota*, *Bacteroidota*, and *Bacillota* phyla than uninfected subjects. *P. acnes* can enhance GC development by producing proinflammatory cytokines such as interleukin (IL)-15, whereas one cytoplasmic fraction of *L. lactis* has been reported to exert an antiproliferative effect on a human stomach cancer cell line, thereby protecting against carcinogenesis; the G0/G1 cell cycle arrest induced by such bacteria was associated with an increase in p53 and p21 expression, a reduction in cyclin D1 expression, and retinoblastoma protein phosphorylation, thereby inducing apoptosis. Thus, it can be suggested that if there is a bacterial species that can promote GC occurrence, the eradication of this bacterium is useful in decreasing GC incidence [21]. Other bacterial genera reported to be increased in GC patients compared to healthy controls include *Enterococcus*, Lactobacillaceae family, *Carnobacterium*, *Glutaminibacter*, and *Fusobacterium* [22].

Thus, the development of GC may be *H. pylori*-independent, since *H. pylori* colonization decreases in later steps of carcinogenesis, whereas other gastric microbial strains predominate, as the gastric microbial community profiles in cancer were reported to significantly differ from those of non-cancerous stomachs. Genera that were more consistently enriched in the GC microbiota belong to intestinal strains, including Lactobacillaceae family, *Streptococcaceae*, *Staphylococcus*, *Clostridium*, and *Fusobacterium nucleatum* [23].

Gastric microbiota balance is extremely complex, and the specific mechanisms causing the occurrence and development of GC remain unknown. The growth of conditional microbiota may inhibit the growth of pathogenic microbes such as *H. pylori* or other pathogenic bacteria such as bacteria with nitrosative functions. Future studies focusing on GC pathogenesis should evaluate *H. pylori* strains, host genetic characteristics, microbiota composition, and environmental factors of the host. Indeed, the mechanisms of microbe–microbe interactions and microbe–host interactions causing GC are multifactorial. Compared with non-GC patients, GC patients have lower alpha diversities and a higher abundances of *H. pylori* [5] but also an overabundance of bacteria that differ in GC patients depending on environmental factors such as the living conditions of patients. Thus, although *H. pylori* is the main trigger of histopathological changes in GC, its relationships with non-*H. pylori* are also involved in the development of GC. Future studies should investigate whether conditional oral or gastric microbiota can serve as hallmarks of GC.

### 2.3. Potential Implications of Other Specific Gastric Bacteria in GC

#### 2.3.1. *Fusobacterium nucleatum*

Interestingly, *Fusobacterium nucleatum* can act directly on host cells affecting the expression of cancer marker genes, thereby promoting the occurrence of cancer, and can indirectly secrete endotoxins to inhibit the immune function of the body and generate an inflammatory microenvironment. *Fusobacterium nucleatum* can activate the nuclear factor-kappa B (NF-κB) pathway to stimulate the production and release of inflammatory cytokines, such as IL-1β, IL-6, IL-8, and tumor necrosis factor (TNF), thereby creating a proinflammatory microenvironment that favors tumor development [24,25]. Moreover, the adhesion of *Fusobacterium nucleatum* from FadA to the E-cadherin of intestinal epithelial cells drives the activation of the Wnt/β-catenin pathway to promote the proliferation of tumor cells [26]. *Fusobacterium nucleatum* has also been reported to be more abundant in the microbiota of GC patients compared with that of non-tumoral controls, and its detection has been associated with GC risk, patient age, tumor size, and decreased survival [27]. Its carcinogenetic mechanism is not fully understood due to its impact on the microenvironment and metabolic function, as well as the deregulation of actin dynamics and changes in cancer cell motility; however, it is accepted that the carcinogenic potential is explicated in the later phases when *H. pylori* is no longer present [28]. Hsieh et al. studied the bacterial species associated with gastric epithelium in 11 GC patients and found that *Fusobacterium nucleatum* was abundantly enriched in GC patients and that the gastric microbes of most GC patients differed from those of non-cancerous gastric disease patients. Analysis of the operating characteristic curve showed that the sensitivity of *Fusobacterium nucleatum* combined with *Clostridium colicanis* and *Fusobacterium canifelinum* in the diagnosis of GC was 100%, and the specificity was about 70% [29].

#### 2.3.2. Lactobacillaceae Family

Lactic acid bacteria, including *Streptococcus*, Lactobacillaceae family, *Bifidobacterium*, and *Lactococcus*, are implicated in carcinogenesis by increasing the amount of toxic N-nitroso compounds and reactive oxygen species (ROS), which, in turn, promote deoxyribonucleic acid (DNA) damage, and by fostering HIF-1-mediated epithelial–mesenchymal transition; then, these bacterial strains can promote the colonization of other carcinogenic pathobionts by inducing immunotolerance. Importantly, lactate itself can act as a fuel source for cancer cell metabolism and can create an immunosuppressive tumor microenvironment by mediating M2-like polarization of tumor-associated macrophages and enhancing the concentration of immune-escaping factors, such as vascular endothelial growth factor and arginase 1. Finally, lactate inhibits T-cell and natural killer cell function and increases the amount of myeloid-derived suppressor cells, which can further suppress natural killer cell cytotoxicity [30,31,32]. The Lactobacillaceae family was reported to act as a carcinogenic factor by producing lactic acid, which may serve as an energy source for tumor cells and stimulate tumor angiogenesis [33]. The Lactobacillaceae family can also enhance the effect of *H. pylori* on human monocyte-derived dendritic cells, leading to dendritic cell maturation and induction, exacerbating the *H. pylori*-mediated inflammatory response, and promoting gastric carcinogenesis [34].

#### 2.3.3. Nitrate Reductase Bacteria

Bacteria implicated in the metabolism of dietary nitrates and nitrites are also considered as potential contributing factors to gastric malignant transformation by increasing the intragastric concentration of nitrite and N-nitroso compounds such as N-nitrosamines and N-nitrosamides [20]. Indeed, N-nitroso compounds are implicated in gastric carcinogenesis, as they generate adducts with DNA, such as O6-methylguanine, which lead to direct DNA damage, and alter the normal intracellular methylation processes, thereby producing epigenetic mutations in some oncogenes [35].

The GC microbiota has been reported to express an increased representation of nitrate reductases, with *Citrobacter*, *Achromobacter*, *Clostridium*, *Campylobacter*, *Deinococcus*, *Sulfurospirillum,* and *Phyllobacterium* representing ascendant species. In another study, *Nitrospirae* was reported to be present in all patients with GC but completely absent in patients with chronic gastritis [36,37].

## 3. Oral Microbiota and GC

The oral microbiota is composed of the microorganisms found in the human oral cavity. Oral cavity bacterial translocation in stomach tissues may produce toxic metabolites and inflammation that can directly damage host cells or interfere with host signaling pathways engaged in cell turnover and survival, thereby increasing the risk of gastric malignant transformation. The overproduction of reactive oxygen species by lactic acid bacteria, as well as N-nitroso compounds, can damage DNA, promote tumor growth and metastasis, and inhibit tumor apoptosis, favoring carcinogenesis. Furthermore, the excessive inflammatory response caused by oral microbiota overabundance can lead to oncogene activation, mutation, DNA damage, cell proliferation, tumor invasion, migration, metastasis, and angiogenesis [38]. A comparative analysis of the gastric microbiota from stomach biopsies of GC patients with dyspeptic controls reported a predominance of *Bacillota*, mainly of oral origin (*Streptococcus*, Lactobacillaceae family, and different *Clostridiales*, such as *Veillonella)* and a lower abundance of *H. pylori*. Among the Bacteroidota phylum, *Prevotella* was the dominant species, and all five classes of *Pseudomonadota (Alpha-*, *Beta-*, *Gamma-*, *Delta-, and Epsilonproteobacteria*), with *Neisseria* and *Haemophilus* as the most dominant genera, were reported to be most present in GC subjects [39]. Wang et al. conducted a case–control study to analyze the difference between GC and chronic gastritis patients and found that the bacterial load in the gastric mucosa was 6.9 × 108 per gram of tissue, with a marked increase in GC cases compared to chronic gastritis controls. Surprisingly, no significant difference was observed between the diversity index of the GC patients and that of patients with chronic gastritis, but an enrichment of five bacterial genera (Lactobacillaceae family, *Escherichia-Shigella*, *Nitrospirae*, *Burkholderia fungorum*, and *Lachnospiraceae*) was found in GC [37]. Hu et al. performed another case–control analysis of oral microbiota, comparing GC patients with healthy controls, and observed that the tongue coatings of patients with GC were significantly thicker than those of healthy controls, with a complex reduced α-diversity in the first group. These findings may promote tongue coating as a potential non-invasive diagnostic tool for early gastric tumors. The authors assessed a significant abundance of *Pseudomonadota* and *Actinomycetota* in GC patients compared with controls, possibly due to lower levels of *Neisseria* and *Haemophilus*. *Fusobacterium* and *Porphyromonas*, which contribute to periodontal disease, were also less common in the GC group, as well as *Prevotella*, *Veillonella*, *Neisseria*, *Lactococcus*, and *Streptococcus*. On the other hand, *Prevotella*, *Streptococcus*, *Actinomyces*, *Veillonella*, and *Leptotrichia* were more abundant in thick-coated GC patients [40]. Another study examined the total bacterial profile of saliva and plaque samples from 50 subjects, including 37 individuals with GC and 13 controls, and evidenced differences in the biomass, species richness, and species diversity between GC patients and normal human subjects not only in the saliva but also in the dental plaque, with a complex overabundance of *Prevotella* spp. and *Aggregatibacter* spp. in the oral cavity of GC patients. The authors reported an increased presence of *Prevotella*, *Porphyromonas*, *Fusobacterium*, *Tannerella*, *Streptococcus*, and *Actinobacillus* in plaque samples of GC patients compared with controls, whereas periodontal pathogens such as *Wolinella* and *Actinomyces* were more common in GC saliva samples, suggesting a relationship between GC and periodontal disease.

This fact might suggest a new potential method for screening suspected GC patients via oral microbiota examination, as periodontal infection can lead to chronic systemic inflammation, which, in turn, represents a risk factor for GC onset [41].

An overabundance of *Corynebacterium* and *Streptococcus*, as well as a reduction in *Haemophilus*, *Neisseria*, *Parvimonas*, *Peptostreptococcus*, *Porphyromonas*, and *Prevotella*, was observed in the oral cavity of GC patients, suggesting that these bacterial strains migrate into the stomach, resulting in dysbiotic overgrowth of pro-inflammatory strains. At the genus level, *Prevotella*, *Neisseria*, *Veillonella*, *Haemophilus*, *Porphyromonas*, *Streptococcus*, *Fusobacterium*, and *Rothia* constitute more than 70% of the salivary microbiota for each histological stage of GC. In particular, pathways involved in isoleucine and valine biosynthesis were highly expressed by the salivary microbiota of GC patients compared to the non-malignant stages, suggesting that alterations in such a metabolism are involved in carcinogenesis [42]. A predisposition to developing GC was also correlated with a different composition of the oral microbiota. An analysis of 16S rRNA genes in the tongue coating microbiota of 57 subjects with GC showed a higher relative abundance of Bacillota and a lower relative abundance of Bacteroidota compared with 80 healthy controls. In particular, a predominance of the genera *Streptomyces*, *Streptococcus*, *Veillonella*, and *Abiotrophia* was reported in GC patients. At the genus level, GC patients have a higher abundance of *Streptomyces*, *Streptococcus*, *Veillonella*, and *Abiotrophia.* In the oral cavity, *Streptococcus* can induce alcohol oxidization to acetaldehyde, a group I human carcinogen. Other Gram-negative bacteria in the oral cavity, such as *Porphyromonas*, *Prevotella*, *Atopobium*, *Ruminococcaceae*, *Stomatobaculum*, *Candidatus Saccharimonas*, *Lachnospiraceae uncultured*, *Oribacterium*, *Eubacterium nodatum group*, *Erysipelotrichaceae*, and *Neisseria* were found to be inversely associated with risk of GC [43]. In another study, a reduction in *Tenericutes*, *M. Orale*, *E. Yurii*, and *Cutibacterium* and increased presence of *BetaPseudomonadota*, *Neisseriales*, *Neisseriaceae*, *N. mucosa*, and *P. pleuritidis* were evidenced in subjects at high risk of GC [44]. Bacteria in the oral cavity can also interact with *H. Pylori*, favoring its overgrowth in the mouth and its migration to the stomach, where it can exert its carcinogenic role; *Candida albicans*, *Fusobacterium nucleatum*, *Porphyromonas gingivalis*, and *Streptococcus mutans* are the most involved strains in this process [45]. Compared with patients affected by other gastric diseases, GC subjects showed major abundance of oropharyngeal commensals such as *Streptococcus*, *Bifidobacterium*, *Escherichia*, *Pseudomonas*, *Neisseria*, *Staphylococcus*, Lactobacillaceae family, *Veillonella*, *Klebsiella*, *Alloprevotella*, *Aggregatibacter*, *Porphyromonas endodontalis*, and *Bacillus* [46]. A recent study by Guo et al. revealed strong co-excluding relationships between the *Helicobacter* genus and multiple potential oral genera in advanced gastric lesions, including *Fusobacterium*, *Neisseria*, *Prevotella*, *Veillonella*, and *Rothia* [16,47].

Specific oral bacterial taxa can be used as GC microbial signatures; among them, *Peptostreptococcus stomatis*, *Streptococcus anginosus*, *Parvimonas micra*, *Slackia exigua*, and *Dialister pneumosintes*, as well as *Clostridium colicanis*, *Fusobacterium canifelinum*, *F. nucleatum*, *Lactobacillus gasseri*, and *Lactobacillus reuteri*, are the most studied strains that may become diagnostics tool for early GC detection in gastric mucosa bioptic samples.

However, in clinical practice, it does not seem feasible to replace traditional screening or diagnostic methods with a gastric microbial examination due to the invasiveness of bioptic sample collection. In this regard, the identification of specific cancer-associated bacterial strains in saliva samples could be used as a diagnostic tool for early GC detection and as a biomarker to predict the overall response rate to pharmacological interventions, avoiding invasive biopsies. Consequently, new strategies to analyze the microbiota composition should be evaluated for screening of both asymptomatic patients at high risk of GC and individual response to treatment; liquid microbial biopsy, consisting of the identification of bacterial circulating DNA, which is similar to genomic material in primary neoplastic tissue, is among such promising techniques [48].

## 4. Gut Microbiota and GC

Recent studies reported an association between intestinal microbiota strains—such as *Clostridium ASF356*, *Lactobacillus ASF361*, *Prevotella copri*, and *Bacillus ASF519*—and GC [34]. For example, analyses of fecal samples from 10 GC patients revealed that Bacteroides were the most important bacteria, followed by *Blautia*, *Veillonella*, and *Sartrella* [49]. In another fecal sample analysis including 20 GC patients and 22 healthy controls, an overabundance of *Shigella*, *Clostridium perfringens*, and *Clostridium*, as well as the depletion of *Bacteroides* and *Bifidobacterium*, characterized the cancer-related microbiota [50]. Recently, Enterobacteriaceae was also identified as a pivotal microbiota strain in all GC subtypes. Moreover, a lower level of intestinal microbiota diversity was correlated with advanced tumor stages in diffuse GC [51]. A recent study sampled paired tumor tissues and fecal samples from 1043 GC and gastritis individuals and found that the relative abundance of *Streptococcus anginosus* was significantly increased in both GC tumor tissues and feces, suggesting that fecal *Streptococcus anginosus* combined with *Streptococcus constellatus* is an accurate and sensitive biomarker for GC [52].

In summary, oral, gastric, and gut dysbiosis may play a pivotal role in gastric carcinogenesis, as summarized in Table 1. The overgrowth of the microbiota mentioned above may partially contribute to the “point of no return” of carcinogenesis after *H. pylori* eradication, suggesting that the modification of the whole microbiota—with a consequent increase in bacterial diversity—is likely beneficial [36].

## 5. Diet: A Potential Therapy to Restore Microbiota Dysbiosis Associated with GC?

Nutritional therapies should be studied thoroughly to delineate their effectiveness in the rebalancing of human microbiota and reducing the progression of carcinogenesis.

### 5.1. Role of SCFAs

In cancer patients, the oral microbiota can transit into the intestinal lumen, where it may be related to increased pH and decreased acid secretion, which, in turn, reduce the efficiency of the gastric barrier, facilitating the orofecal transit of bacteria. A reduction in short-chain fatty acid (SCFAs) producer microbes as a consequence of oral dysbiosis in the gut microbiota may be a contributing factor in GC development and progression. In fact, under normal conditions, SCFAs exert a protective effect on the cell cycle, apoptosis, and immune response by modulating cellular pathways (Akt/mTOR and MEK/ERK signaling pathways) and transcription factors (downregulation of NF-κB), as well as epigenetic regulation (inhibition of histone deacetylase inhibitor (HDAC)–histone deacetylases activity, DNA methylation, histone phosphorylation, and methylation) [53]. In particular, in GC cells, butyrate produces beneficial alterations in the proliferation of apoptosis-related genes, decreasing the expression of focal adhesion kinase and increasing the expression of DAPK1/2, which induces apoptosis. Furthermore, it can stimulate the p53-p21 pathways, leading to cell cycle arrest and apoptosis of cancerous cells, suggesting a potential adjuvant role of these SCFAs in chemotherapy for GC [54]. In addition, a study conducted by Yuan-Linag et al. revealed a depletion of pathways (acetyl-CoA fermentation to butanoate II, L-glutamate degradation V, and 4-aminobutanoate degradation V) associated with SCFA production in GC patients compared with healthy controls, indicating the existence of a more inflammatory microenvironment and dysbiotic microbial communities in GC mucosal samples [47]. Another study demonstrated that the SCFA concentration can be measured in peripheric plasma samples; plasma concentrations of most SCFAs were found to be lower in patients with GC than in gastritis patients. Moreover, other intermediates of tricarboxylic acid were demonstrated to differ in the cancer population relative to non-cancer controls, suggesting them as potential GC diagnostic markers in peripheral blood [55]. In a recent case–control study conducted by Nouri et al., a reduced concentration of SCFAs was observed in GC patients compared with healthy controls, which was correlated with oral microbiota dysbiosis, suggesting that alterations in the composition of the oral microbiota can contribute to a reduction in SCFAs. In particular, the authors observed a negative correlation between the carcinogenic *Streptococcus*, *Abiotrophia*, and *Leuconostoc* strains and the concentration of total SCFAs, which, in turn, may contribute to the promotion of the proinflammatory TNFAIP8 and IL-6/STAT3 pathways, favoring cancer onset and progression. Therefore, the authors suggested that SCFA administration may represent an early intervention and targeted treatment against GC, although more evidence is needed before testing this hypothesis in clinical practice [56]. In another study, it was observed that butyric acid produced by *Porphyromonas gingivalis* exerted an inhibiting effect on *H. pylori* growth and proliferation in the oral cavity, suggesting that the bactericidal properties of this SCFA make it suitable for use as a preventive therapy against *H. pylori* translocation in the stomach and, therefore, against GC genesis. Conversely, butyric acid has been shown to exhibit an antimicrobial effect on *Campylobacter* species, *Escherichia coli*, and *Staphylococcus aureus*, which have also been associated with GC onset and progression [57].

Furthermore, SCFAs represent potential adjuvant chemotherapeutic agents. Indeed, a recent study of a combination treatment with butyrate and cisplatin showed an increased apoptosis rate in GC cell lines and an in vivo xenograft tumor model, as well as reduced cell migration and invasion [58].

SCFA composition is also influenced by surgical resection of the stomach. A recent study analyzing the 16SrRNA gene sequence in stool samples of 20 GC patients after subtotal gastrectomy demonstrated a general reduction in beneficial SCFA production after surgery. The latter may further increase the permeability of the intestinal mucosal barrier and negatively affect postoperative recovery. This is probably due to an overall increase in *Bacteroidota* and a decrease in *Pseudomonadota* and *Actinomycetota* and, at the genus level, higher abundances of *Streptococcus*, *Escherichia/Shigella*, *Akkermansia*, *Verrucomicrobia*, *Dialister*, *Prevotella*, and *Veillonella* in the gut [50]. A recent study conducted by Castano-Rodriguez et al. observed an increased richness and phylogenetic diversity but not Shannon’s diversity in GC patients when compared to dyspeptic controls. Among the bacterial taxa, *Lactococcus*, *Veillonella*, *Fusobacterium*, and *Leptotrichia* were reported to be increased only in GC samples. The authors also highlighted that the bacterial carbohydrate metabolism was enriched in GC patients and; as carbohydrate digestion and absorption are partly responsible for SCFA production, this suggested that alteration in energy metabolism may be directly involved in carcinogenesis [33]. Hu et al. reported a complex depletion of pathways (acetyl-CoA fermentation to butanoate II, L-glutamate degradation V, and 4-aminobutanoate degradation V) associated with SCFA production in GC, indicating the existence of a more inflammatory microenvironment and dysbiotic microbial communities in GC [47].

### 5.2. Prebiotics

Prebiotics—including inulin, fructo-oligosaccharide (FOS), and galacto-oligosaccharides (GOS)—are fibers promoting the growth of specific groups of anaerobic colonic indigenous bacteria; they are undigestible by endogenous enzymes in the small intestine but are actively fermented by colonic bacteria, selectively promoting the growth of beneficial bacteria [59]. To date, although scientific interest in the role of prebiotics in the course of cancer is growing, original studies remain lacking. One recent interesting study showed that some carrot compounds, such as acetylenic oxylipins like falcarinol and falcarinol, can inhibit cell growth in different cancers, such as breast cancer, colon cancer, and lymphoid leukemia. Nevertheless, the protective effect against cancer seems to be due to intrinsic anti-inflammatory and proapoptotic properties rather than a modulatory effect on microbiota [60,61,62]. The effect of inulin consumption, a natural dietary soluble fiber consisting of a mixture of oligo- and/or polysaccharides should be studied, given its prebiotic potential, technological properties, and beneficial effects on the gut microbiota [63].

After gastrectomy for GC, a short course of enteral nutrition (EN) is recommended as support to preserve intestinal structure and function and enhance intestinally mediated immunity. However, postoperative complications such as diarrhea can negatively affect the overall recovery of postoperative patients with GC [64]. Diarrhea is a common complication of EN, which affects recovery and prolongs the length of hospital stay. Considering that intestinal flora imbalance is associated with a reduction in protective *Bifidobacteria* and an increase in the counts of pathogens, administration of fiber prebiotics may represent a potential treatment for EN-induced diarrhea. A recent study of 120 GC patients divided into three groups (fiber-free nutrition formula (FF group, *n* = 40), fiber-enriched nutrition formula (FE group, *n* = 40), and fiber- and probiotic-enriched nutrition formula (FEP group, *n* = 40)) demonstrated that the combination of fiber and probiotics was significantly effective in treating EN-associated diarrhea in such patients. Fiber can be metabolized by intestinal flora and produce SCFAs, which are the preferred source of energy for colonic cells to improve gut barrier integrity and function. Moreover, some fermentation of fiber in the colon may favor the selective growth of beneficial bacteria such as *Bifidobacteria* and Lactobacillaceae family [65]. Furthermore, in animal models, perioperative immunopurified with supplementation of SCFAs has been proposed to reinforce the strength of gastrointestinal anastomosis, thereby reducing the risk of surgical-site infection [66].

### 5.3. Polyphenols

The positive effect of polyphenols may be related to the induction of apoptosis and cell cycle arrest, as well as to the inhibition of proinflammatory mediators [67]. Polyphenols act as antioxidant, anti-inflammatory, immunomodulatory, and gastroprotective agents by interacting with oral, gastric, and intestinal bacteria in a beneficial way [68].

A recent meta-analysis conducted by Ma et al. demonstrated that supplementation with polyphenol can significantly improve the production of health-promoting bacterial species like *Bifidobacterium* and Lactobacillaceae family and suppress the production of harmful or undesirable bacterial species like *Clostridium* and *Escherichia coli* [69]. Among polyphenols, the most important studied compounds are green tea, resveratrol, curcumin, and quercetin [70,71,72,73,74]. In particular, promising preclinical studies have been conducted to demonstrate the antiproliferative, antimetastatic, and chemotherapeutic roles of resveratrol in inhibiting GC growth, either directly or indirectly by increasing the amount of beneficial *Ruminococcus*, *Akkermansia*, *Dehalobacterium*, and *Anerostipes* in the gut. However, more evidence is needed before these compounds can be recommended as effective anti-GC adjuvant therapy in clinical practice [75,76,77,78,79].

Conversely, tryptophan, a dietary compound processed by Bacillota (*Clostridium sporogenes*, *Ruminococcus gnavus*, and Lactobacillaceae family) has been shown to play an essential role in the suppression of anticancer immune responses, in enhancing ROS production and DNA damage, and in increasing the malignant properties of cancer tissues, leading to tumor spread [80]. In conclusion, even though preclinical studies and one American clinical trial evaluating the effect of eicosapentaenoic acid’s role in liver metastases in colorectal cancer have shown promising results, demonstrating the effective anticancer role of polyphenols, at present, these compounds are not used as antitumoral therapies in clinical practice to modulate microbiota [81].

### 5.4. Fermented Foods and Probiotics

Probiotics are the living organisms in our gut that contribute to healthy conditions. They are found in fermented foods such as kefir, sauerkraut, yogurt, and kimchi. Natural yogurt, sweetened yogurt, and mature cheese are the most consumed among fermented dairy foods. *L. casei* is used in milk fermentation processes to produce yogurt and cheeses, and it has been associated with important health-promoting benefits, such as regulation of the intestinal microbiota, tumor inhibition, proapoptotic and antiproliferative effects, and the production of bioactive peptides in fermented milk. Among fermented foods, yogurt can be beneficial with respect to gastrointestinal diseases by acting positively on the gut microbiota, balancing inflammation and dysbiosis; however, a recent meta-analysis of 16 international studies found no association between yogurt intake and GC risk, suggesting that its potential beneficial probiotic role in GC development can be achieved only with much higher levels of intake [82,83]. Importantly, the role of lactic acid bacteria in GC seems to be bimodal. In vitro and in vivo evidence has focused on the role of lactic acid bacteria in the production of ROS, causing DNA damage and harmful N-nitroso compounds, which are implicated in mutagenesis, angiogenesis, protooncogene expression, and inhibition of apoptosis. Lactate, a lactic acid bacteria metabolite, is also a fuel for cancer cell survival [84].

After gastrectomy, gastric acid in the gastrointestinal tract is almost suppressed, causing modifications in the environment and the composition of the gut microbiota. Increased migration of pathogens can damage the intestinal mucosal immune barrier and provoke intestinal and systemic inflammation, which, in turn, delays the patient’s postoperative recovery. Therefore, it has been hypothesized that probiotic administration may positively affect the composition of the gut microbiota, thereby rebalancing gastrectomy-induced dysbiosis and favoring the recovery of the patient [85]. Dietary enrichment with probiotics (*Bifidobacterium* spp., Lactobacillaceae family, and *Streptococcus* spp.) in a murine model resulted in a decrease in the polyamine concentration, in association with anticancer effects, by fostering gastric cancer cell apoptosis [86]. In a recent randomized double-blind trial, oral supplementation with probiotic *C. butyricum*, an anaerobic Gram-positive bacillus, after gastrectomy for GC resulted in a reduced inflammatory response in terms of leucocyte and neutrophil count and the expression of proinflammatory cytokines such as IL-1b, IL-6, and TNF-a, as well as in amelioration of immune and nutritional status in terms of immunoglobulin, lymphocyte, albumin, and total protein levels. These results are probably due to the increased production of SCFAs, such as acetic acid, propionic acid, butyric acid, and isobutyric acid, facilitating postoperative recovery and decreasing the risk of complications. After *C. butyricum* administration, an overabundance of beneficial intestinal bacteria, such as *Bacteroides*, *Faecalibacterium*, and *Gemmiger*, with a concomitant reduction in pathogenic *Streptococcus*, *Desulfovibrio*, and *Actinomyces*, was also reported [87]. Another study analyzing the effect of probiotics (*B. infantis*, *L. acidophilus*, *Enterococcus faecalis*, and *Bacillus cereus*) after gastrectomy demonstrated a decrease in inflammatory response and an improvement in the immune index, with an overall promotion of postoperative recovery [88]. Moreover, a recent preclinical study suggested that probiotic administration (e.g., of butyrate-producing bacteria and *Bifidobacterium*) after endoscopic GC resection can reduce the risk of tumor recurrence [89]. Another important consideration is that different methods of digestive tract reconstruction produce variable effects on the microbial composition. 16S rRNA analysis revealed that fecal microbiota transplantation in gastrectomized mice with the fecal microbiota of Roux-en-Y reconstruction patients can reverse dysbacteriosis triggered by radical gastrectomy and elevate the relative abundance of some SCFA-producing bacteria, thereby ameliorating postoperative nutritional status, colitis, and overall recovery of GC subjects. A 16SrRNA analysis of fecal bacteria of RY patients revealed an overabundance of *Bacteroides*, *Clostridium*, and *Ruminococcus*, which are noted SCFA producers [90].

### 5.5. Dietary Patterns

Imbalanced dietary habits with increased consumption of smoked, salty foods and reduced intake of fiber, vegetables, and fruits have been associated with an increased risk of gastric carcinogenesis [91]. As food additives in processed meats, the consumption of nitrates, nitrites, and N-nitrosodimethylamine was reported to result in in an increased risk of GC [92]. N-nitroso compounds can be derived by the diet (essentially processed meat and smoked fish) or by endogenous synthesis from pathogenic bacterial strains such as *Veillonella*, *Staphylococcus*, *Neisseria*, *Clostridium*, *Haemophilus*, Lactobacillaceae family, and *Nitrospirae*; such compounds have been demonstrated to be overabundant in GC patients, favoring the initiation and progression of carcinogenesis [93]. Recent evidence suggests a potential carcinogenic role of lipids, cholesterol, and bile acids in different types of neoplasms, as they contribute to inflammation-mediated tumor growth through oncogenic activation and negatively interact with the host microbiota, favoring procarcinogenic dysbiosis. Nevertheless, specific studies on their role in GC are still lacking [94].

A recent scoping review evidenced that a more versatile high-fat diet was associated with a higher rate of intestinal microbial dysbiosis, as well as with increased GC risk, as it led to an overabundance of Lactobacillaceae family reaching the stomach. Conversely, a high intake of fruit, vegetables, dairy products, and seafood presented a protective microbial signature and an inverse correlation with GC [95].

Even though considerable evidence have confirmed that Western dietary habits are correlated with worse prognoses in different types of cancer, no study has focused on the specific effects of diet on the oral microbiota in gastric cancer development and progression [96].

Nutritional strategies such as fasting and caloric restriction have been evaluated as adjuvant treatments in different cancers, as it was observed that they can protectively modulate systemic metabolism. In an ongoing clinical trial (NCT01642953), researchers are restricting patient diets after gastric cancer surgery to assess whether fasting promotes recovery and reduces mortality and adverse events. However, the role of such dietary habits with respect to gastric and intestinal microbiota composition still needs to be elucidated [97,98].

## 6. Conclusions

**Figure 1:**
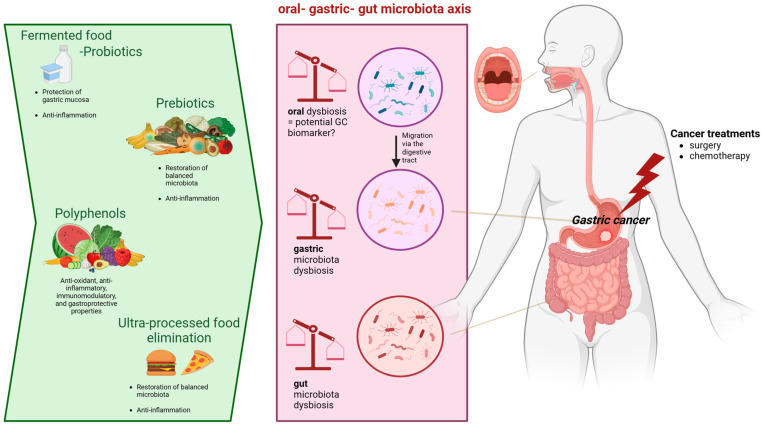
Potential nutritional interventions that can positively influence the oral–gastric–gut microbiota during gastric cancer treatment.

This review highlights that the oral microbiota is a pivotal influencer in terms of tumor development and response to gastric anticancer treatments. Although a large body of preclinical evidence has been released, the exact mechanism whereby the altered oral microbiota contributes to carcinogenesis remains unknown. The oral microbiota, as well as gastric and/or gut bacteria, can be used a potential marker for the diagnosis and prognosis of GC. Moreover, the adjustment of microbiota composition using efficient dietary strategies may improve clinical GC patient outcomes. Further studies on the microbiota in GC patients could allow us to understand the mechanisms of GC pathogenesis and propose promising oral–gastric gut microbiota interventions to optimize GC treatments, including the use of prebiotics, probiotics, and nutritional interventions. In this context, Figure 1 illustrates nutritional interventions such as prebiotics, fermented food, probiotics, or other dietary strategies that may regulate the composition and balance of the oral–gastric–gut microbiota environment. For these reasons, more attention should be paid to the effect of nutritional interventions on the microbiota of GC patients during the course of treatment.

## Figures and Tables

**Table 1:** Microbiota compositional changes and potential effects on GC carcinogenesis mechanisms.

	Microbiota Compositional Changes	Mechanisms of Carcinogenesis	References
Oral microbiota	↑ *Veillonella* ↑ *Neisseria* ↑ *Haemophilus* ↑ *Porphyromonas* ↑ Bacillota ↓ Bacteroides	Periodontal infection → gastric chronic inflammation and dysbiosis ↑ N-nitroso compounds	[40,41,42,43]
	↑ *Streptococcus*	↑ Induction of alcohol to be oxidized to acetaldehyde, a group I human carcinogen	[45]
	↑ *Candida albicans*, *Fusobacterium nucleatum*, *Porphyromonas gingivalis,* and *Streptococcus mutans*	↑ Interaction *H. pylori* → ↑ *H. pylori* survival in the unsuitable environment of the mouth, fostering *H. pylori* migration to the stomach and exertion of its carcinogenic role	[45]
Gastric microbiota	↑ *H. pylori* ↑ Gastric dysbiosis	Oncoprotein cytotoxin-associated gene A Destabilization of cellular junctions via disruption of the E-cadherin-β-catenin complex Activation of proliferating pathways such as the ERK-MAP kinase cascade Oncogenic transformation of gastric cells via vacuolation of cytotoxin A Promotion of abnormal cell survival Inflammatory effect on the gastric mucosal surface of the stomach with altered production of mucin	[12,13,14,15,16]
	↑ Nitrate reductase bacteria	↑ Nitrate reductase activity ↑ Proinflammatory activity	[36,37]
	↑ *Streptococcus*, Lactobacillaceae family, and *Lactococcus*	↑ N-nitroso compounds ↑ Reactive oxygen species Promotion of DNA damage	[30,31,32,33]
	↓ *Lactococcus lactis*	↓ Antiproliferative activity in a human stomach cancer cell line ↑ Cell apoptosis	[21]
	↑ *Fusobacterium nucleatum* ↓ Bacterial diversity	↑ NF-κB, IL-1β, IL-6, IL-8, TNF, and FadA	[22,24,25,26,27,28]
Gut microbiota	↑ Enterobacteriaceae ↓ *Bifidobacterium* ↓ Bacteroides ↑ *Shigella* ↑ *Clostridium perfringens* ↓ Bacterial diversity	↓ Production of SCFAs and their protective effects on the cell cycle, apoptosis, and immune stimulation, impacting the Akt/mTOR and MEK/ERK signaling pathways ↓ Inhibition of NF-κB	[50,51,52,53]

Abbreviations: ↑, increase; ↓, decrease; Akt/mTOR, protein kinase B/mammalian target of rapamycin; DNA, deoxyribonucleic acid; ERK-MAP, extracellular signal-regulated kinase–mitogen-activated protein kinase; *H. pylori*, *Helicobacter pylori*; NF-κB, nuclear factor kappa-light-chain-enhancer of activated B cells; IL, interleukin; SCFAs, short-chain fatty acids; TNF, tumor necrosis factor.

## Data Availability

Not applicable.
